# PATIENT PATHWAYS TO UGANDA’S FIRST SPECIALISED EARLY INTERVENTION IN PSYCHOSIS SERVICE AND RELATION TO THEIR CLINICAL OUTCOMES

**DOI:** 10.64898/2026.04.30.26352152

**Published:** 2026-05-01

**Authors:** Emmanuel Kiiza Mwesiga, Wilber Ssembajjwe, Rossette Immy Ndigamanya, Sophia Balinga, Blessed Tabitha Aujo, Mary Ampaire, Andrea Kaggwa Kaddu, Andrew Sentoogo Ssemata, Allan Kalungi, Ronald Kiguba, Johesephat Byamugisha, Mark Kaddumukasa, Martha Sajatovic, Noeline Nakasujja

**Affiliations:** 1. Department of Psychiatry, College of Health Sciences, Makerere University; 2. Specialised Treatment Early in Psychosis Service at Makerere University Hospital.; 3. MRC/ UVRI & LSHTM Uganda Research Unit, Entebbe, Uganda.; 4. Butabika National Referral Mental Hospital; 5. Mental Health Department, Soroti Regional Referral Hospital.; 6. Rhodium Mental Health Specialists Hospital; 7. Department of Pharmacy, College of Health Sciences, Makerere University; 8. Directorate of Health Services, Makerere University; 9. Department of Internal Medicine, College of Health Sciences, Makerere University; 10. Neurological and Behavioural Outcomes Centre, University Hospital Case Medical Centre, Case Western Reserve University

**Keywords:** First-episode psychosis, early intervention, pathways to care, sub-Saharan Africa, traditional healers, symptom remission, quality of life, referral systems

## Abstract

**Background:**

Early Intervention for Psychosis Services (EIPS) enhance outcomes for individuals experiencing their first episode of psychosis (FEP). However, in low-resource settings, there is limited knowledge about i) the pathways patients take to access EIPS, ii) the proportion and factors associated with acceptance of referral to EIPS, and iii) if different pathways to EIPS services affect clinical outcomes. Uganda’s first EIPS, the Specialised Treatment Early in Psychosis Service at Makerere University Hospital (STEP_MaKH), presents a unique opportunity to explore these important questions.

**Aims:**

We aimed to examine the pathways to EIPS, the factors associated with referral to specialised psychosis care and the impact of initial treatment-seeking behaviour on longterm symptom remission and quality of life.

**Methods:**

We conducted a multiple-method study. Pathways to care were assessed retrospectively using the WHO Encounter Form among adults with FEP eligible for referral to STEP_MaKH. Among those who completed referral and enrolled in STEP_MaKH. Symptom severity and quality of life were followed prospectively for 12 months. Modified Poisson regression identified predictors of referral completion. Kaplan–Meier methods and Cox proportional hazards models examined time to symptom remission and time to achieving a good quality of life.

**Results:**

Of the 187 adults with first-episode psychosis eligible for referral to STEP_MaKH, Native/religious healers (n = 86) were the predominant first point of contact. Only 56 (29.9%) accepted referral to STEP_MaKH. Participants referred from Mulago National Referral Hospital more likely to enrol than those referred from Butabika (RR = 4.7; 95% CI: 2.90–7.87). Longer delays from first treatment contact were associated with reduced likelihood of reaching STEP_MaKH (RR = 0.99 per month; p = 0.041). After enrolment, symptoms improved rapidly with 60% achieving PANSS remission by Month 1, and fewer than 10% remained non-remitted by Months 2–3. In adjusted Cox models, participants initially seen by mental health workers achieved remission more quickly than those initially seen by non-medical personnel (HR = 1.48; 95% CI: 1.05–2.10). Older age was associated with slower remission (HR = 0.94; p = 0.023). Quality of life improved over the follow-up period, with earlier attainment of good quality of life among those initially managed by mental health workers.

**Conclusions:**

Pathways to care for FEP in Uganda are complex and culturally mediated, with substantial attrition before specialised early psychosis care is reached. Referral completion is strongly shaped by referral site and by delays in the care pathway. Once in specialised care, clinical outcomes improve rapidly, and initial contact with mental health workers is associated with faster symptom remission and earlier gains in quality of life. Strengthening referral systems, reducing pathway delays, and developing collaborative detection-and-referral links with community and frontline providers are key priorities for optimising early psychosis outcomes in low-resource settings.

## Introduction and Background:

First-episode psychosis (FEP) refers to the initial presentation of psychotic symptoms, typically characterised by delusions, hallucinations, disorganised thinking, and behavioural disturbances occurring for the first time in an individual’s life (1–3). This phase represents a critical and potentially reversible period in the trajectory of psychotic disorders, during which timely intervention is associated with improved symptomatic, functional, and quality-of-life outcomes (2, 4). Unlike chronic schizophrenia, FEP is marked by greater neurobiological plasticity, lower cumulative treatment exposure, and a higher probability of remission, making early detection and intervention particularly consequential (5, 6). Delays during this stage, often conceptualised as duration of untreated psychosis (DUP), are strongly associated with poorer long-term prognosis (6, 7).

It is recommended that patients experiencing their first-episode psychosis (FEP) receive initial care in an early intervention for psychosis service (EIPS) (6, 8, 9).Evidence-based interventions, including low-dose second-generation antipsychotics, psychotherapy, and supported education and employment, improves outcomes in FEP Treatment of FEP in EIPS is associated with better outcomes, including quality of life, duration of hospitalization and symptom remission in high income countries (10–14). The effectiveness of EIPS has shifted policy frameworks globally, positioning early intervention as a cornerstone of modern psychosis care.

The pathways patients take to reach EIPS vary widely and are influenced by health system structures, cultural beliefs, and healthcare accessibility (15, 16). In high-income settings, studies have mapped these pathways, demonstrating that early access to specialised care reduces the duration of untreated psychosis and improves clinical and functional outcomes (8, 17).

Despite strong evidence from high-income settings, the implementation and evaluation of EIPS in low- and middle-income countries (LMICs), particularly in sub-Saharan Africa, remain limited. The few available studies in many low-resource settings, particularly in sub-Saharan Africa, document that pathways to care are often non-linear, and shaped by cultural explanatory models, stigma, and constrained mental health infrastructure. Patients often seek for help from multiple providers, including traditional healers and general medical services, before reaching psychiatric care (18–23). Delays in accessing appropriate treatment contribute to poorer long-term outcomes, underscoring the need to understand referral dynamics in these settings (24–26). Empirical data on how patients reach EIPS and whether these pathways influence clinical outcomes in Sub-Saharan Africa are scarce.

Understanding patient trajectories, defined as the sequence and transitions between care providers from symptom onset to specialised treatment, is essential for evaluating real-world access to and effectiveness of EIPS. Care pathways are not merely descriptive phenomena; they influence delays, treatment exposure, family responses, and ultimately clinical outcomes. Mapping trajectories allows identification of system bottlenecks, attrition points, and inequities in referral mechanisms (15, 17). In emerging EIPS models within LMICs, analysing trajectories is particularly important to distinguish between service efficacy and access limitations.

Given the scarcity of data on EIPS is Uganda and SSA more broadly, we conducted a retrospective descriptive analysis of clinical care pathways taken by 187 patients with first episode psychosis over a 12-month time-frame within a health care system in Uganda. We describe the referral pathways to Uganda’s first EIPS, the Specialised Treatment Early in Psychosis Service at Makerere University Hospital (STEP_MaKH), and examine their impact on quality of life and symptom remission. By mapping patient trajectories, we aim to1) examine the pathways to EIPS, 2) the factors associated with referral to specialised psychosis care and 3) the impact of initial treatment-seeking behaviour on long-term symptom remission and quality of life. Understanding these dynamics is critical for designing interventions that reduce delays and improve engagement with EIPS.

## Methods:

### Study design:

The study employed a multiple-method design, with retrospective assessments to determine pathways to care and prospective measures to examine relationships between different pathways with quality of life and symptom remission among 187 patients with FEP during their first year of treatment in a Ugandan health system.

### Study setting:

Mental health services in Uganda are delivered through a tiered system comprising primary care facilities, general hospitals, and specialised psychiatric hospitals. Public specialist psychiatric care is concentrated in a limited number of referral institutions, including Butabika National Referral Mental Hospital, while integration of mental health into general medical services remains variable. Access to care is influenced by workforce shortages, stigma, geographical barriers, and pluralistic health-seeking practices, with many individuals initially consulting traditional or religious healers prior to engaging with formal psychiatric services (27, 28). Mulago National Referral Hospital and Butabika National Referral Mental Hospital serve as major entry points for patients with severe mental illness, including first-episode psychosis.

Participants were recruited from Butabika National Referral Mental Hospital, Mulago National Referral Hospital, and affiliated clinical services. Individuals presenting with symptoms consistent with first-episode psychosis were screened for eligibility using diagnostic criteria and inclusion/exclusion parameters. Eligible participants underwent retrospective assessment of pathways to care. All eligible individuals were offered referral to STEP_MaKH. Participants who accepted referral and completed enrolment entered the prospective follow-up cohort and were assessed longitudinally over 12 months for symptom severity and quality-of-life outcomes.

STEP_MaKH is a pioneering mental health service in Uganda, established in 2024, that aims to promote the early identification, intervention, and ongoing care of individuals experiencing their first episode of psychosis (FEP) (29)(ref). It was established to address critical gaps in early psychosis care in Uganda. The clinic offers specialised assessment, diagnosis, and treatment. Care is delivered by a team that includes psychiatrists, clinical psychologists, and psychiatric nurses, each with a small patient caseload. The model emphasises family involvement, psychoeducation, psychopharmacology, and, where feasible, psychosocial interventions. The clinic primarily receives referrals from Butabika and Mulago National Referral Hospitals. STEP_MaKH is increasingly integrating measurement-based care tools, including components from EPINET’s Core Assessment Battery, to monitor patient progress, guide treatment decisions, and enhance quality improvement (30, 31).

### Participants:

The study included first-episode psychosis patients operationalised as having a confirmed psychosis disorder according to the MINI International Neuropsychiatric Inventory (MINI)(32). The sample size of 187 participants included in the analysis, were adults aged 18–60, never having been treated with antipsychotic medication or on medication for less than six weeks’ duration and no substance use disorder, HIV/AIDS or syphilis.

### Variables and data sources:

Pathways to care were identified using the World Health Organisation’s Encounter form to systematically gather information on the sources of care utilised by participants before approaching a mental health professional (33). Participants were then offered a referral to STEP_MaKH. Outcome measures were collected only among participants enrolled in STEP_MaKH and were administered as part of routine measurement-based care at baseline (enrolment) and at Months 1, 2, 3, 6, and 12. Participants who agreed to referral to STEP_MaKH received routine specialised care, including repeated measures to track outcomes from the EPINET core assessment battery (34, 35). The battery includes the six-item Positive and Negative Signs and Symptoms of Schizophrenia (PANSS-6) scale to assess symptom severity and the EPINET Quality of Life Tool to assess quality of life. These have been described elsewhere.

## Statistical analysis:

Categorical variables such as sex, religion, district of residence, employment status, and type of first contact were summarized using frequencies and percentages. Continuous variables, including age, were summarized using means and standard deviations. To compare participants who received specialized psychiatric care (STEP_MaKH) with those who did not, Pearson’s Chi-square tests were used for categorical variables and independent-sample t-tests were used for continuous variables. Fisher’s exact tests were applied where expected cell counts were small. Statistical significance was set at p < 0.05.

A multivariate modified Poisson regression model with robust standard errors was fitted to identify factors independently associated with referral completion to the Specialised Treatment Early in Psychosis Service (STEP_MaKH). Independent variables included demographic characteristics, recruitment site, and type of first contact. Adjusted risk ratios (RRs) with 95% confidence intervals (CIs) were reported. Care-seeking trajectories were examined using the World Health Organisation (WHO) Encounter Form, which captures sequential points of contact across different providers. Pathways were categorised by type of first contact (traditional or religious healer, general hospital, police, social worker, or psychiatric service) and mapped to final presentation at Butabika National Referral Mental Hospital (BNRMH) or STEP_MaKH. The number and proportion of individuals following each unique pathway were calculated and summarised separately for the overall sample, those enrolled in EIPS (STEP_MaKH), and those who remained at BNRMH. Visual flow diagrams were used to illustrate common referral patterns and identify variations in access to psychiatric care.

Longitudinal changes in clinical outcomes were analysed for participants followed at STEP_MaKH. The outcomes were plotted across four time points (t_0_ to t_3_) and stratified by initial point of contact, traditional/religious healers versus medical and mental health providers. These visualisations were used to describe differences in trajectories of symptom remission and quality of life over time. Finally, survival analyses were performed to assess the time to PANSS remission and achieving satisfactory quality of life (defined as QOL ≥7 - defined by principal component analysis). Kaplan–Meier survival curves were generated to estimate the cumulative probability of achieving satisfaction over time. Covariate-adjusted hazard ratios (HRs) were obtained using Cox proportional hazards models, with age, sex, BMI, and PANSS included as predictors. Model diagnostics were assessed using log-rank tests and the proportional hazards assumption. All statistical analyses were conducted using Stata version 18 (StataCorp LLC, College Station, TX, USA) and SAS 9.4M6

### Ethics and Dissemination.

Ethical clearance was obtained from the School of Medicine Research and Ethics Committee (SOMREC) (Mak-SOMREC-2024–869) and institutional approval from Butabika National Referral Mental Hospital and Makerere University Hospital. All participants provided informed consent prior to participation.

### Results:

A total of 187 participants were recruited for the study, with 85.5% (160/187) recruited from Butabika National Referral Mental Hospital. The median age of the participants was 28.9 years, and most (60%) were males. Most participants were from Wakiso (46%) and Kampala (31%), with smaller proportions from Mukono and other districts. The majority were Christian (81%), followed by Muslims (17%), and a small number from other religions. In terms of education, most had completed primary (35%) or secondary (39%) education, while smaller numbers had tertiary (22%) or no formal education (5%)

### Pathways to care:

This figure illustrates the multiple, often cyclical care-seeking paths individuals take before reaching formal psychiatric services. Native/religious healers (n = 86) are the predominant first point of contact. Notably, a large proportion of participants used general psychiatric services as their initial point of care for the first episode of psychosis (n = 61). Other initial points of contact were established with social workers (n = 1), police (n = 7), and general hospitals (n = 30). Following that initial contact, the patients continued to seek care from various service providers, including traditional or religious healers. For example, of the 86 individuals who first received care from native/religious healers, 27 continued care with another native/traditional healer, 11 went to a general hospital, 38 to a psychiatric hospital, and one accepted to continue care with STEP_MaKH. Other pathways to specialised care are shown in the figure below

Referral to STEP_MaKH: The pathways to STEP-MaKH were not direct for some individuals. Although more than half of the participants reached STEP_MaKH via a single referral, some required two or three referrals, as shown in the figure below. For example, the participant who first contacted the police required three referrals to reach STEP_MaKH. The one participant whose initial contact was a social worker never made it to the specialised clinic.

Only 56 of the 187 participants agreed to a referral to the STEP_MaKH. Participants who accepted referral to STEP_MaKH were slightly younger, with a mean age of 27.2 years (SD = 7.9), compared with 30.5 years (SD = 9.4); this difference was statistically significant (p = 0.023). There were also statistical differences among those who reached STEP_MaKH by referral source and educational level. Other comparisons are shown in the table below. Among those who received specialised care, most were from Butabika National Referral Hospital (32/56; 57.1%). However, a higher proportion of participants (16/18, 88.9%) accepted referral to STEP_MaKH from Mulago NRH compared to only 32/160 (20.0%) from Butabika Hospital. Further distribution of participants who accepted referral compared to those who didn’t across various sociodemographic characteristics are shown below.

In the multivariate modified Poisson regression model, the predictors of accepting referral to STEP_MaKH included the referral site, with participants referred from Mulago National Hospital four times more likely to make it to STEP_MaKH than those from Butabika National Referral Mental Hospital (RR 4.7 (2.90;7.87, P<0.001). Furthermore, the person initially contacted and the time since contact were also associated with a reduced risk of presenting to STEP_MaKH, as shown in the table below.

## Initial point of care and patient outcomes.

### Symptom remission (PANSS-6 score <14):

At baseline, mean PANSS scores were highest among participants initially seen by mental health workers, followed by those seen by non-medical personnel and general medical doctors. Between baseline and Month 2, all groups showed a substantial reduction in mean PANSS scores, with values declining to approximately 7.4–7.8 across groups. Further reductions were observed at Month 3 and Month 6. At Month 6, mean PANSS scores were lowest across the follow-up period, at approximately 6.3 for participants initially seen by mental health workers, 6.5 for those seen by general medical doctors, and 6.7 for those seen by non-medical personnel. By Month 12, mean PANSS scores among participants initially seen by mental health workers continued to decline slightly, while scores among those initially seen by general medical doctors remained relatively stable and scores among those initially seen by non-medical personnel increased modestly

Over the follow-up period, the cumulative probability of remaining non-remitted declined in all groups. Participants initially seen by mental health workers exhibited the lowest survival probabilities (i.e., the highest cumulative incidence of remission) at each follow-up time point, followed by those initially seen by general medical doctors, whereas participants initially seen by non-medical personnel had the highest probability of remaining non-remitted. Divergence between the curves was observed early in follow-up and persisted across subsequent time points, although the probability of non-remission was low in all groups by the end of follow-up

## Adjusted Cox proportional hazards model for predictors of time to symptom remission

In the adjusted Cox proportional hazards model, the initial care provider was associated with time to PANSS remission. Compared with participants initially seen by non-medical personnel, those initially managed by mental health workers had a higher rate of remission (adjusted HR = 1.48; 95% CI: 1.05–2.10; p = 0.026). Participants initially seen by general medical doctors did not differ significantly from those seen by non-medical personnel (adjusted HR = 1.12; 95% CI: 0.78–1.61; p = 0.540). Baseline symptom severity was strongly associated with remission; higher baseline PANSS scores were associated with a lower rate of remission (adjusted HR = 0.91 per point increase; 95% CI: 0.87–0.95; p < 0.001). Increasing age was also associated with a slower rate of remission (adjusted HR = 0.94 per year; 95% CI: 0.91–0.97; p = 0.023). Sex and body mass index were not significantly associated with time to PANSS remission

### Quality of life:

Mean quality-of-life (QOL) scores increased over time across all three initial care provider groups. At baseline, mean QOL scores were similar across groups, ranging from approximately 5.6 to 5.7. By Month 2, QOL scores had increased across all groups, with participants initially seen by non-medical personnel showing slightly higher mean scores than those initially seen by mental health workers or general medical doctors. Between Month 2 and Month 3, a marked increase in mean QOL was observed among participants initially seen by mental health workers, reaching the highest mean QOL score at Month 3. Continued improvement was observed through Month 6 across all groups, with mean QOL scores converging at approximately 7.2–7.6. By Month 12, mean QOL scores remained highest among participants initially seen by mental health workers, followed by those initially seen by general medical doctors, while participants initially seen by non-medical personnel had slightly lower mean scores

### Time-to-Event Analysis:

Kaplan–Meier curves were used to describe time to achieving good quality of life (defined as QOL ≥ 7) by initial care provider. Across follow-up, the probability of not yet achieving good QOL declined in all groups. Participants initially seen by mental health workers consistently showed the lowest probability of remaining below the QOL threshold, followed by those initially seen by general medical doctors, while participants initially seen by non-medical personnel had the highest probability of remaining below the threshold.

### Cox Proportional Hazards Model:

A Cox regression model was fitted with age, sex, BMI, PANSS, as covariates. Hazard ratios (HR) greater than 1 indicate faster achievement of satisfaction; HR less than 1 indicate slower time. Compared with participants initially seen by non-medical personnel, those initially managed by mental health workers had a higher rate of achieving good QOL (adjusted HR = 1.36; 95% CI: 1.02–1.82; p = 0.033). Participants initially seen by general medical doctors did not differ significantly from those initially seen by non-medical personnel (adjusted HR = 1.10; 95% CI: 0.82–1.48; p = 0.540). Higher baseline QOL scores were associated with a faster transition to good QOL (adjusted HR = 1.22 per point increase; 95% CI: 1.10–1.35; p < 0.001), while higher baseline PANSS scores were associated with a slower transition (adjusted HR = 0.94 per point increase; 95% CI: 0.90–0.98; p = 0.004). Age, sex, and body mass index were not significantly associated with time to achieving good QOL.

**Table:** 

Covariate	Adjusted HR	95% CI	p-value
Initial care provider			
Mental health worker	1.36	1.02 – 1.82	0.033
General medical doctor	1.1	0.82 – 1.48	0.54
Non-medical personnel	Reference		
Baseline QOL score (per point)	1.22	1.10 – 1.35	<0.001
Baseline PANSS score (per point)	0.94	0.90 – 0.98	0.004
Age (per year)	0.99	0.96 – 1.02	0.52
Male sex (vs female)	0.97	0.72 – 1.31	0.85
BMI (per kg/mÂ^2^)	1	0.98 – 1.02	0.91

## Discussion

This study provides the first empirical examination of referral pathways and their impact on patient outcomes in individuals with a first episode of psychosis to Uganda’s first Early Intervention in Psychosis Service, the Specialized Treatment Early in Psychosis Clinic at Makerere University Hospital (STEP_MaKH). Three key findings emerged. First, help-seeking trajectories were highly variable and often began outside the formal health system, particularly with traditional and religious healers. Second, fewer than one-third of eligible patients accepted referral to specialised early psychosis care, with referral site, initial care provider, and delays from first contact strongly predicting referral completion. Third, although clinical outcomes improved substantially over 12 months for all participants, outcomes were better when initial contact was with a mental health worker. These findings have important implications for scaling early psychosis care in low-resource settings.

### Pathways to care:

Pathways were highly variable and frequently began outside formal psychiatric care. Nearly half of the participants first sought help from traditional or religious healers, while others entered care through general psychiatric services, general hospitals, or (less commonly) the police or social services. This pattern is consistent with evidence across sub-Saharan Africa showing that explanatory models of illness, stigma, access barriers, and health-system fragmentation produce non-linear, multi-contact trajectories before specialist care is reached (18, 20, 21, 26). Importantly, the pathway diagrams also illustrate “cycling” across provider types, suggesting that delays are not simply due to late first help-seeking, but to repeated transitions between providers before effective linkage to specialised services.

### Referral Patterns:

There was substantial attrition before STEP_MaKH. Fewer than one-third of eligible patients completed referral, and predictors were predominantly system- and pathway-level features rather than basic demographics. Referral site was the strongest determinant, with referral completion being substantially higher at Mulago than at Butabika. The unacceptably long delay in returning of mental health services to Mulago Hospital after renovation may be the reason why there was greater referral. Initial contact at a general hospital was also associated with reduced probability of enrolling at STEP_MaKH, plausibly reflecting competing care pathways, limited structured referral mechanisms, or weak continuity across facility types. Beyond the site, longer delays from first contact reduced the likelihood of reaching STEP_MaKH, indicating that time itself functions as an attrition mechanism (27, 36). Possible explanations include families becoming demoralised, resources being depleted, symptoms becoming chronic, or patients stabilising sufficiently in non-specialist settings that onward referral is deprioritised (37–39). In the adjusted model, these results suggest that scaling EIPS in Uganda could be improved by strengthening referral systems and the reliability of linkages, particularly in high-volume psychiatric settings where most patients first present.

### Pathways and Clinical Outcomes:

Patients initially seen by mental health providers on average had more severe illness severity. This is in keeping with previous literature from this setting, highlighting how patients with more severe psychosis are sent to psychiatric services (40). Once patients entered STEP_MaKH, outcomes improved rapidly, with initial provider type showing measurable associations with speed of improvement. Irrespective of initial provider type, symptom severity declined rapidly, with 60% achieving PANSS remission (scores < 14) by Month 1 and very low non-remission by Months 2–3. This is similar to high-income literature highlighting the potential of specialised early psychosis care even in low-resource contexts when a functional pathway into care exists (14, 26). However, initial contact with a mental health worker was associated with a shorter time to PANSS remission than contact with non-medical personnel, and mental health worker contact similarly predicted earlier attainment of good QOL. The general medical doctor pathway did not differ significantly from non-medical personnel in either model, suggesting that the benefit is not simply “any medical contact,” but may reflect earlier recognition of psychosis by trained mental health providers. Baseline severity strongly predicted slower remission, indicating that early-stage severity remains a key driver of trajectory even after specialised care begins (41, 42). Older age predicted slower remission, which may reflect longer untreated illness, more entrenched functional impairment, differential family responses, or unmeasured comorbidity (43, 44). For quality of life, higher baseline QOL accelerated attainment of good QOL, while higher baseline PANSS slowed it, reinforcing the clinical logic that symptom burden and functional recovery remain tightly coupled (45, 46).

### Implications for Early Intervention in Low-Resource Settings:

Improving early psychosis outcomes in Uganda and across sub-Saharan Africa requires integrating clinical evidence, system design, and practical implementation. STEP_MaKH demonstrates that specialized early intervention services produce tangible benefits in symptom resolution and quality of life, even in resource-constrained settings. Their effectiveness depends critically on the pathways that lead to them. Differences in referral completion rates between Mulago and Butabika hospitals reveal high-impact opportunities for system optimization: standardized referral protocols, rapid family orientation at point of referral, warm handoffs with scheduled appointments, and bidirectional feedback loops that enable referring providers to track outcomes and sustain referral behaviour. These mechanisms shorten the time between first help-seeking and specialist linkage. This system optimization has been done in existing early intervention services and networks including EPINET, Australian Early Psychosis Collaborative Consortium (AEPCC) and Early intervention services in the UK (35, 47, 48). Similar services must be adapted to low resource settings in Africa. Since traditional and religious healers often serve as the first point of contact, they must be engaged as partners rather than obstacles (22, 23). This may be through co-developed referral agreements, culturally sensitive psychoeducation, and shared communication channels that facilitate timely transitions to formal care. For sustainable scale-up, early intervention programs must be embedded within national mental health strategies and existing health system structures, not developed in isolation. However, coordinating nodes for various services are required. Other practical implementation steps include: adopting digital referral-tracking systems to identify bottlenecks in real time, building capacity among frontline mental health workers to accelerate both linkage and clinical recovery, aligning program placement with institutional readiness and community demand, and establishing measurement-based care platforms that support ongoing quality improvement (49–51).

### Future Directions:

Three research priorities will advance early psychosis care in sub-Saharan Africa. First, establish standardized measurement across diverse contexts. Multisite cohorts spanning varied health systems and cultural settings are needed to move beyond single-site findings. Standardized tools like the Nottingham Onset Schedule (NOS-DUP) should measure duration of untreated psychosis, enabling clearer interpretation of treatment delays and meaningful comparison with global benchmarks (52). Second, understand the human factors shaping care pathways. Qualitative research with patients, caregivers, traditional healers, and frontline clinicians should examine how cultural beliefs, stigma, economic barriers, and trust in different care providers influence help-seeking decisions and pathway navigation. This work must identify not just barriers but also existing strengths in community support systems that can be leveraged. Third, test implementation strategies through pragmatic trials. Rather than demonstrating efficacy alone, studies should rigorously evaluate scalable interventions including digital referral tracking, community-based psychoeducation co-designed with traditional healers, task-shifting models for frontline workers, and measurement-based care protocols. Their effectiveness should be compared across different facility types and referral networks. These trials should measure not only clinical outcomes but also implementation metrics: referral completion rates, time to first specialist contact, cost per patient linked, and sustainability beyond external funding. In all these phases, the inclusion of people with lived experience will ensure the interventions are acceptable and useful (53). Together, this research agenda will generate the context-specific evidence needed to reduce treatment delays and expand access at scale.

### Strengths and Limitations:

A major strength of this study is its use of structured instruments, such as the WHO Encounter Form, PANSS-6, and WHO-QOL, to capture both pathway dynamics and longitudinal clinical outcomes. By focusing on Uganda’s first EIPS, the study provides novel data from a context where such services are still in their early stages of development. However, several limitations warrant caution. The sample size for patients enrolled at STEP_MaKH was modest, limiting statistical power for some outcomes. Pathway reconstruction relied on retrospective reporting, raising the possibility of recall bias. The study did not directly measure DUP using validated instruments such as the NOS-DUP; however, DUP is likely a key mediator of the observed associations between pathways and outcomes. Finally, findings reflect patterns in a specific urban referral context and may not generalise to rural settings or other regions of Uganda.

### Conclusion:

In Uganda, as in much of sub-Saharan Africa, pathways to care in patients with psychotic disorders vary and are culturally mediated. However, empirical evidence linking these pathways to health-system attrition and downstream outcomes has been limited. This study provided some answers by examining referral completion into STEP_MaKH alongside 12-month clinical trajectories after enrolment. Our findings show that specialised early intervention can produce rapid clinical gains, but that its population impact depends on whether patients can reliably reach it early enough for those gains to matter. The dominant bottleneck is referral completion, which was low and was shaped primarily by the referring site and by cumulative delays from first contact. Once patients entered specialised care, such as STEP_MaKH, outcomes improved rapidly. However, earlier contact with mental health workers predicted faster remission and earlier quality-of-life gains, suggesting that pathway “quality” and not just pathway “length,” influences outcomes. Importantly, these results reposition scale-up as an implementation problem requiring standardised protocols at key entry points, active linkage and navigation for families, and targeted capacity building for frontline mental health workers. These interventions may reduce attrition, shorten delays, and accelerate recovery in real-world African health systems.

## Figures and Tables

**Figure 1:**
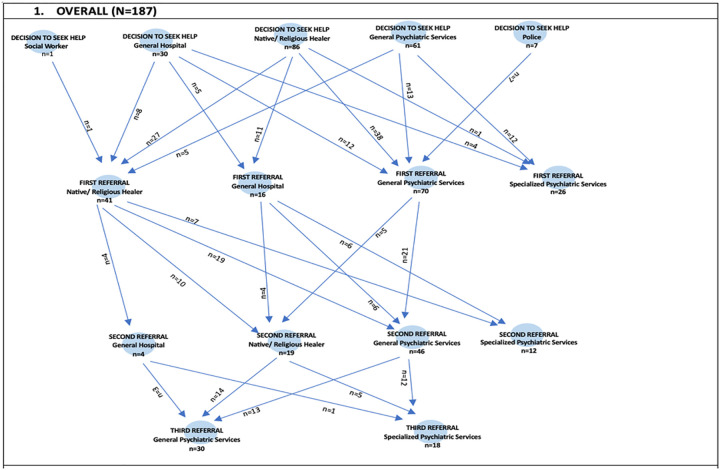
Pathways to care for the whole sample.

**Figure 2:**
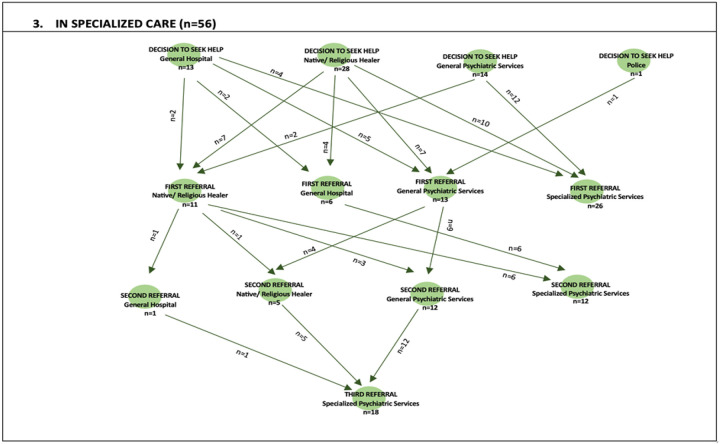
Pathways to care to STEP_MaKH

**Figure 3:**
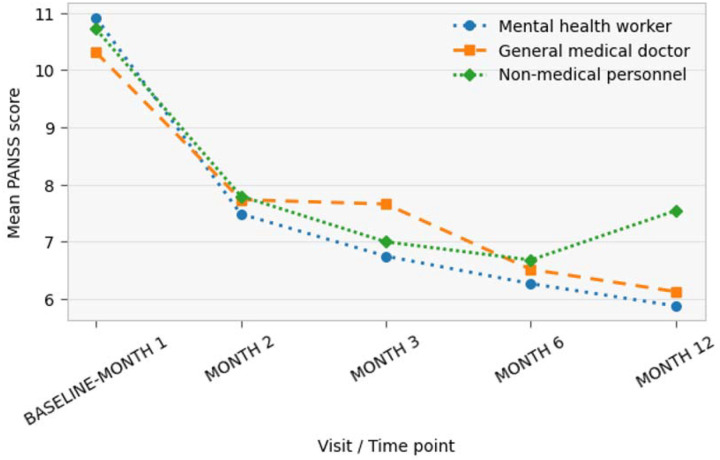
Changes in PANSS Scores Over Time by Initial Care Provider

**Figure 4:**
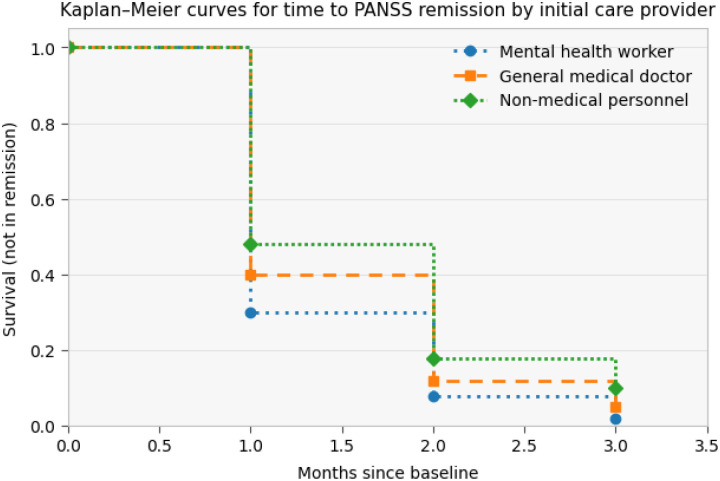
Time to symptom improvement by initial provider

**Figure 5:**
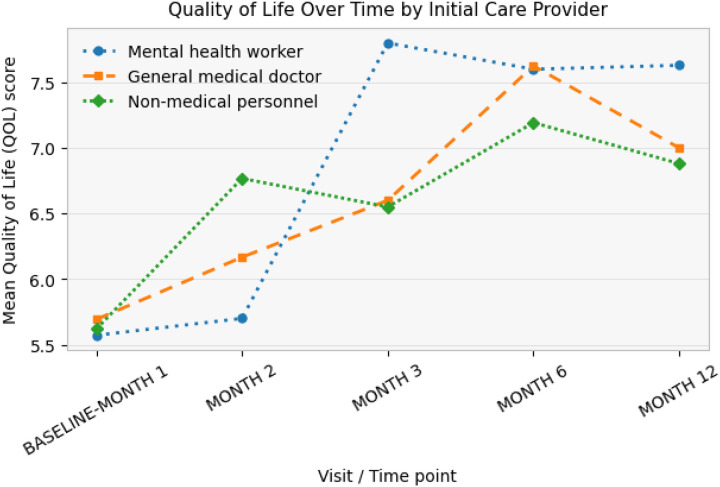
Quality of life changes over time by initial care provider.

**Figure 6:**
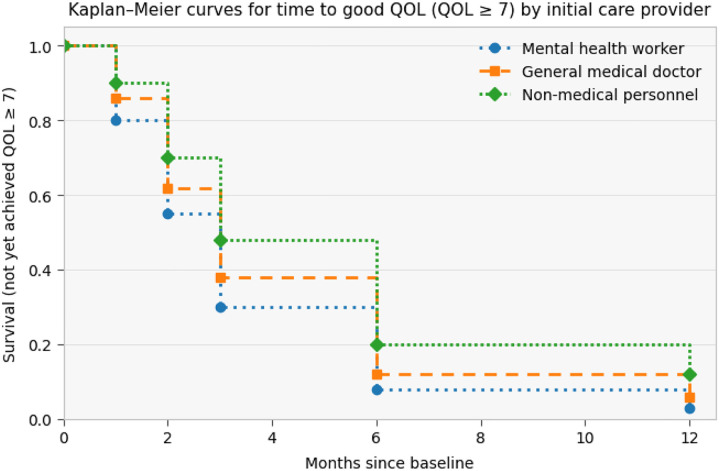
KM graph for time to good quality of life by initial care provider.

**Table 1:** Demographic Characteristics

Factor	Level	IN SPECIALIZED CARE		p-value
		No (n=131)	Yes (n=56)	
Hospital	Butabika NRH	128 (97.7%)	32 (57.1%)	< 0.001
	Mulago NRH	2 (1.5%)	16 (28.6%)	
	Other	1 (0.8%)	8 (14.3%)	
Age	Mean (SD)	30.5 (9.4)	27.2 (7.9)	0.023
Sex	Male	83 (63.4%)	29 (51.8%)	0.139
	Female	48 (36.6%)	27 (48.2%)	
District	Kampala	40 (30.5%)	19 (33.9%)	0.065
	Wakiso	55 (42.0%)	31 (55.4%)	
	Mukono	14 (10.7%)	1 (1.8%)	
	Other	22 (16.8%)	5 (8.9%)	
Religion	Christian	106 (80.9%)	46 (82.1%)	<0.001
	Muslim	24 (18.3%)	6 (10.7%)	
	Other	1 (0.8%)	4 (7.1%)	
Education level	No formal education	12 (9.2%)	2 (3.6%)	0.017
	Primary	51 (38.9%)	13 (23.2%)	
	Secondary	48 (36.6%)	23 (41.1%)	
	Tertiary	20 (15.3%)	18 (32.1%)	
Employment status	Unemployed	86 (65.6%)	40 (71.4%)	0.440
*Do you work for a pay outside your home?*	Employed	45 (34.4%)	16 (28.6%)	
Average monthly salary (n=61)	1–100,000	16 (35.6%)	3 (18.7%)	0.072
	100,001–500,000	22 (48.9%)	6 (37.5%)	
	500,001–1,000,000	3 (6.7%)	5 (31.2%)	
	Above 1,000,000	4 (8.9%)	2 (12.5%)	
How long was the initial contact (in Median (IQR) months) Range		6(1; 36) 0– 420	5 (1; 24) 0 – 240	0.471

**Table 2:** Results for fitting a multivariate modified Poisson model

Factor	Level	ADJUSTED MODEL RR (95% CI)	p-value
Hospital			< 0.001
	Butabika NRH	Reference	
	Mulago NRH	4.7 (2.90; 7.87)	
	Other	4.03 (2.47; 6.58)	
Age	Per year increase	0.99(0.97; 1.02)	0.915
Sex	Male	Reference	0.183
	Female	1.32 (0.87; 2.01)	
District			0.426
	Kampala	Reference	
	Wakiso	1.16 (0.75; 1.78)	
	Mukono	0.34 (0.06; 1.71)	
	Other	0.96 (0.42; 2.16)	
Religion			0.408
	Christian	Reference	
	Muslim	0.63 (0.30; 1.33)	
	Other	1.15 (0.62; 2.15)	
Education level			0.055
	No formal education	Reference	
	Primary	1.00 (0.28; 3.50)	
	Secondary	1.56 (0.45; 5.41)	
	Tertiary	2.20 (0.63; 7.62)	
Employment status			0.811
	Unemployed	Reference	
	Employed	0.93 (0.55; 1.59)	
Initial contact for help			< 0.001
	Native/Religious healer	Reference	
	Police	0.71 (0.14; 3.50)	
	Social worker	-	
	General hospital	0.55 (0.34; 0.88)	
	Psychiatric Services	0.65 (0.38; 1.11)	
How long ago was the initial contact			0.041
	Per month increase	0.99 (0.99; 0.99)	

**Table 3:** Predictors to symptom remission

Covariate	Adjusted HR	95% CI	p-value
**Initial care provider**			
Mental health worker	**1.48**	**1.05 – 2.10**	**0.026**
General medical doctor	1.12	0.78 – 1.61	0.540
Non-medical personnel	Reference		
Baseline PANSS score (per point)	0.91	0.87 – 0.95	<0.001
Age (per year)	0.94	0.91 – 0.97	0.023
Male sex	1.01	0.58 – 1.76	0.981
BMI (per kg/m^2^)	1.00	0.98 – 1.02	0.921

## References

[1] Boydell KM, Stasiulis E, Volpe T, Gladstone B. A descriptive review of qualitative studies in first episode psychosis. Early intervention in psychiatry. 2010;4(1):7–24. 20199476 10.1111/j.1751-7893.2009.00154.x

[2] Malla A, Payne J. First-episode psychosis: psychopathology, quality of life, and functional outcome. Schizophrenia bulletin. 2005;31(3):650–71. 16006593 10.1093/schbul/sbi031

[3] Reed SI. First-episode psychosis: A literature review. International journal of mental health nursing. 2008;17(2):85–91. 18307596 10.1111/j.1447-0349.2008.00515.x

[4] Ran MS, Xiao Y, Zhao X, Zhang TM, Yu YH, Mao WJ, Family history of psychosis and outcome of people with schizophrenia in rural China: 14-year follow-up study. Asian J Psychiatr. 2018;32:14–9. 29197709 10.1016/j.ajp.2017.11.016

[5] Farooq S, Fonseka N, Ali MW, Milner A, Hamid S, Sheikh S, Early Intervention in Psychosis and Management of First Episode Psychosis in Low- and Lower-Middle-Income Countries: A Systematic Review. Schizophr Bull. 2024;50(3):521–32. 38525604 10.1093/schbul/sbae025 PMC11059814

[6] Üçok A. Treatment Principles of First-Episode Psychosis. Noro Psikiyatr Ars. 2021;58(Suppl 1):S12–s6. 34658630 10.29399/npa.27424 PMC8498813

[7] Penttilä M, Jääskeläinen E, Hirvonen N, Isohanni M, Miettunen J. Duration of untreated psychosis as predictor of long-term outcome in schizophrenia: systematic review and meta-analysis. The British Journal of Psychiatry. 2014;205(2):88–94. 25252316 10.1192/bjp.bp.113.127753

[8] Murphy BP, Brewer WJ. Early intervention in psychosis: clinical aspects of treatment. Advances in Psychiatric Treatment. 2018;17(6):408–16.

[9] Gouveia M, Costa T, Morgado T, Sampaio F, Rosa A, Sequeira C. Intervention Programs for First-Episode Psychosis: A Scoping Review Protocol. Nursing Reports. 2023;13(1):273–83. 36810277 10.3390/nursrep13010026 PMC9944946

[10] Kane JM, Schooler NR, Marcy P, Correll CU, Brunette MF, Mueser KT, The RAISE early treatment program for first-episode psychosis: background, rationale, and study design. J Clin Psychiatry. 2015;76(3):240–6. 25830446 10.4088/JCP.14m09289 PMC7477907

[11] Azrin ST, Goldstein AB, Heinssen RK. Early intervention for psychosis: the recovery after an initial schizophrenia episode project. Psychiatric Annals. 2015;45(11):548–53.

[12] Larsen TK, Melle I, Auestad B, Haahr U, Joa I, Johannessen JO, Early detection of psychosis: positive effects on 5-year outcome. Psychological medicine. 2011;41(7):1461–9. 20942996 10.1017/S0033291710002023

[13] Fusar-Poli P, Salazar de Pablo G, Correll CU, Meyer-Lindenberg A, Millan MJ, Borgwardt S, Prevention of Psychosis: Advances in Detection, Prognosis, and Intervention. JAMA Psychiatry. 2020;77(7):755–65. 32159746 10.1001/jamapsychiatry.2019.4779

[14] Williams R, Ostinelli EG, Agorinya J, Minichino A, De Crescenzo F, Maughan D, Comparing interventions for early psychosis: a systematic review and component network meta-analysis. eClinicalMedicine. 2024;70. 10.1016/j.eclinm.2024.102537 PMC10955207 38516103

[15] O’Connell N, O’Connor K, McGrath D. Early Intervention in Psychosis services: A systematic review and narrative synthesis of the barriers and facilitators to implementation. 2021;65(1):e2. 10.1192/j.eurpsy.2021.2260 PMC8792869 34913421

[16] Powell T, Glozier N, Conn K, Einboden R, Buus N, Caldwell P, The impact of early intervention psychosis services on hospitalisation experiences: a qualitative study with young people and their carers. BMC Psychiatry. 2024;24(1):350. 38730333 10.1186/s12888-024-05758-4 PMC11088060

[17] Tiller J, Maguire T. Early intervention in psychosis services: A systematic review and narrative synthesis of barriers and facilitators to seeking access. 2023;66(1):e92. 10.1192/j.eurpsy.2023.2465 PMC10755576 37929296

[18] Esan O, Appiah-Poku J, Othieno C, Kola L, Harris B, Nortje G, A survey of traditional and faith healers providing mental health care in three sub-Saharan African countries. Social psychiatry and psychiatric epidemiology. 2019;54(3):395–403. 30456425 10.1007/s00127-018-1630-y

[19] Lilford P, Wickramaseckara Rajapakshe OB, Singh SP. A systematic review of care pathways for psychosis in low-and middle-income countries. Asian J Psychiatr. 2020;54:102237. 33271678 10.1016/j.ajp.2020.102237

[20] Galvin M, Chiwaye L, Moolla A. Perceptions of causes and treatment of mental illness among traditional health practitioners in Johannesburg, South Africa. South African Journal of Psychology. 2023;53(3):403–15. 38037643 10.1177/00812463231186264 PMC10688254

[21] Berhe KT, Gesesew HA. Traditional healing practices, factors influencing to access the practices and its complementary effect on mental health in sub-Saharan Africa: a systematic review. 2024;14(9):e083004. 10.1136/bmjopen-2023-083004 PMC11429370 39322598

[22] Abbo C, Ekblad S, Waako P, Okello E, Musisi S. The prevalence and severity of mental illnesses handled by traditional healers in two districts in Uganda. African Health Sciences. 2009;9(2). PMC2890991 20589155

[23] Abbo C. Profiles and outcome of traditional healing practices for severe mental illnesses in two districts of Eastern Uganda. Global health action. 2011;4(1):7117. 10.3402/gha.v4i0.7117 PMC3150106 21845144

[24] Vacheron MN, Veyrat-Masson H, Wehbe E. [What support of young presenting a first psychotic episode, when schooling is being challenged?]. L’Encephale. 2017;43(6):570–6. 10.1016/j.encep.2017.10.001 29128195

[25] Teshager S, Kerebih H. Pathways to psychiatric care and factors associated with delayed help-seeking among patients with mental illness in Northern Ethiopia: a cross-sectional study. 2020;10(7):e033928. 10.1136/bmjopen-2019-033928 PMC7383954 32713844

[26] Farooq S, Fonseka N, Ali MW, Milner A. Early Intervention in Psychosis and Management of First Episode Psychosis in Low- and Lower-Middle-Income Countries: A Systematic Review. 2024;50(3):521–32. 10.1093/schbul/sbae025 PMC11059814 38525604

[27] Wakida EK, Talib ZM, Akena D, Okello ES, Kinengyere A, Mindra A, Barriers and facilitators to the integration of mental health services into primary health care: a systematic review. Systematic reviews. 2018;7(1):211. 30486900 10.1186/s13643-018-0882-7 PMC6264616

[28] Mwesiga EK, Ssemata AS, Nakitende AJ, Ongeri L, Semeere A, Loewy R, Exploring facilitators for a transition from alternative and complementary therapies to evidence-based treatments in Ugandan first-episode psychosis patients. medRxiv. 2022:2022.02.21.22270378.

[29] MakChs. Launch of the Early Intervention Psychiatry Services at Makerere University Hospital | Makerere University College Of Health Sciences 2025 [updated 2026. Available from: https://chs.mak.ac.ug/news/launch-early-intervention-psychiatry-services-makerere-university-hospital.

[30] Goldman MB, Janicak PG. Early interventions for psychosis. Current Psychiatry. 2021;20(10):13.

[31] Bello I, Lee R, Malinovsky I, Watkins L, Nossel I, Smith T, OnTrackNY: The development of a coordinated specialty care program for individuals experiencing early psychosis. Psychiatric services. 2017;68(4):318–20. 27973999 10.1176/appi.ps.201600512 PMC5846122

[32] Lecrubier Y, Sheehan D, Hergueta T, Weiller E. The mini international neuropsychiatric interview. European Psychiatry. 1998;13(1004):198s–s. 10.1016/S0924-9338(97)86748-X 19698595

[33] Organization WH. Organization of services for mental health. 2003.

[34] George P, Rosenblatt A, Daley T, Ghose SS, O’Brien J, Rajapaksa S, , editors. The early psychosis intervention network (EPINET) national core assessment battery: building the foundation for a learning health care partnership. the American Public Health Association’s VIRTUAL Annual Meeting and Expo American Public Health Association; 2020.

[35] Niendam TA. 9.4 EPINet: A National Approach for Data-Driven Improvement in Early Psychosis Care. Journal of the American Academy of Child & Adolescent Psychiatry. 2022;61(10):S291.

[36] Atewologun F, Adigun OA. A comprehensive review of mental health services across selected countries in sub-Saharan Africa: assessing progress, challenges, and future direction. 2025;5(1):49. 10.1007/s44192-025-00177-7 PMC11977099 40195169

[37] Gagiu C, Dionisie V, Manea MC, Covaliu A, Vlad AD, Tupu AE, Quality of Life in Caregivers of Patients with Schizophrenia: A Systematic Review of the Impact of Sociodemographic, Clinical, and Psychological Factors. Behavioral Sciences. 2025;15(5):684. 40426466 10.3390/bs15050684 PMC12109056

[38] Andualem F, Melkam M, Tadesse G, Nakie G, Tinsae T, Fentahun S, Burden of care among caregivers of people with mental illness in Africa: a systematic review and meta-analysis. BMC Psychiatry. 2024;24(1):778. 39511520 10.1186/s12888-024-06227-8 PMC11542449

[39] Hunt X, Abdurahman H, Omobowale O, Afolayan A, Munetsi E, Dzapasi L, Interventions for adolescents and adults with psychosis in Africa: a systematic review and narrative synthesis. Global Mental Health. 2022;9:223–40. 36618745 10.1017/gmh.2022.25 PMC9806991

[40] Mwesiga EK, Nakasujja N, Nakku J, Nanyonga A, Gumikiriza JL, Bangirana P, One year prevalence of psychotic disorders among first treatment contact patients at the National Psychiatric Referral and Teaching Hospital in Uganda. PLoS One. 2020;15(1):e0218843. 31995567 10.1371/journal.pone.0218843 PMC6988969

[41] Kaminga AC, Dai W, Liu A, Myaba J, Banda R, Wen SW, Rate of and time to symptomatic remission in first-episode psychosis in Northern Malawi: A STROBE-compliant article. Medicine. 2018;97(45):e13078. 30407306 10.1097/MD.0000000000013078 PMC6250544

[42] van Dee V, Schnack HG, Cahn W. Systematic review and meta-analysis on predictors of prognosis in patients with schizophrenia spectrum disorders: An overview of current evidence and a call for prospective research and open access to datasets. Schizophrenia Research. 2023;254:133–42. 36863229 10.1016/j.schres.2023.02.024

[43] Majuri T, Haapea M, Nordström T, Säynäjäkangas V, Moilanen K, Tolonen J, Effect of onset age on the long-term outcome of early-onset psychoses and other mental disorders: a register-based Northern Finland Birth Cohort 1986 study. European child & adolescent psychiatry. 2024;33(6):1741–53. 37568059 10.1007/s00787-023-02279-5 PMC11211101

[44] Catalan A, Salazar de Pablo G, Aymerich C, Guinart D, Goena J, Madaria L, ”Short” Versus “Long” Duration of Untreated Psychosis in People with First-Episode Psychosis: A Systematic Review and Meta-Analysis of Baseline Status and Follow-Up Outcomes. Schizophrenia Bulletin. 2024;51(5):1206–30. 10.1093/schbul/sbae201 PMC12414564 39580760

[45] Watson P, Zhang J-P, Rizvi A, Tamaiev J, Birnbaum ML, Kane J. A meta-analysis of factors associated with quality of life in first episode psychosis. Schizophrenia Research. 2018;202:26–36. 30005933 10.1016/j.schres.2018.07.013

[46] DeRosse P, Nitzburg GC, Blair M, Malhotra AK. Dimensional symptom severity and global cognitive function predict subjective quality of life in patients with schizophrenia and healthy adults. Schizophrenia Research. 2018;195:385–90. 29056491 10.1016/j.schres.2017.10.018 PMC5908765

[47] Thompson A, Fitzsimons J, Killackey E, Ahern S, Amminger P, Alvarez-Jimenez M, The Australian Early Psychosis Collaborative Consortium (AEPCC): Improving Clinical Care in Early Psychosis. Australas Psychiatry. 2023;31(3):306–8. 37171091 10.1177/10398562231174691

[48] Neale A, Kinnair D. Early intervention in psychosis services. Br J Gen Pract. 2017;67(661):370–1. 28751348 10.3399/bjgp17X692069 PMC5519118

[49] Kabukye JK, Namagembe R, Nakku J, Kiberu V, Sjölinder M, Nilsson S, Implementing a Hospital Call Center Service for Mental Health in Uganda: User-Centered Design Approach. JMIR Hum Factors. 2024;11:e53976. 38843515 10.2196/53976 PMC11190627

[50] Nixdorf R, Kotera Y, Baillie D, Garber Epstein P, Hall C, Hiltensperger R, Development of the UPSIDES global mental health training programme for peer support workers: Perspectives from stakeholders in low, middle and high-income countries. PloS one. 2024;19(2):e0298315. 38408108 10.1371/journal.pone.0298315 PMC10896522

[51] Kohrt BA, Mutamba BB, Luitel NP, Gwaikolo W, Onyango Mangen P, Nakku J, How competent are non-specialists trained to integrate mental health services in primary care? Global health perspectives from Uganda, Liberia, and Nepal. Int Rev Psychiatry. 2018;30(6):182–98. 30810407 10.1080/09540261.2019.1566116 PMC6499679

[52] Singh SP, Cooper JE, Fisher HL, Tarrant CJ, Lloyd T, Banjo J, Determining the chronology and components of psychosis onset: The Nottingham Onset Schedule (NOS). Schizophrenia research. 2005;80(1):117–30. 15978778 10.1016/j.schres.2005.04.018

[53] Law H, Morrison AP. Recovery in psychosis: a Delphi study with experts by experience. Schizophrenia bulletin. 2014;40(6):1347–55. 24727194 10.1093/schbul/sbu047 PMC4193718

